# Integration of Motor Proteins – Towards an ATP Fueled Soft Actuator

**DOI:** 10.3390/ijms9091685

**Published:** 2008-09-04

**Authors:** Akira Kakugo, Kazuhiro Shikinaka, Jian Ping Gong

**Affiliations:** Graduate School of Science, Hokkaido University, Sapporo 060-0810, Japan

**Keywords:** Biological motors, self-assembly, hierarchical structure, soft-bio-machine

## Abstract

We present a soft bio-machine constructed from biological motors (actin/myosin). We have found that chemically cross-linked polymer-actin complex gel filaments can move on myosin coated surfaces with a velocity as high as that of native F-actin, by coupling to ATP hydrolysis. Additionally, it is shown that the velocity of polymer-actin complex gel depends on the species of polycations binding to the F-actins. Since the design of functional actuators of well-defined size and morphology is important, the structural behavior of polymer-actin complexes has been investigated. Our results show that the morphology and growth size of polymer-actin complex can be controlled by changes in the electrostatic interactions between F-actins and polycations. Our results indicate that bio actuators with desired shapes can be created by using a polymer-actin complex.

## 1. Introduction

There are two basic differences between the motion in a man-made machine and in a biological motor. One is in their principles. The motion of a man-made machine, which is constructed from hard and dry materials such as metals, ceramics or plastics, is realized by the relative displacement of the macroscopic constituent parts of the machine. In contrast to this, the motion of a living organism, which consists of soft and wet protein and tissues, is caused by a molecular deformation that is integrated to a macroscopic level through its hierarchical structure [1–3]. The other is in their energy sources. The man-made machine is fueled by electrical or thermal energy, which is used with an efficiency of around 30%, but a biological motor is driven by direct conversion of chemical energy with much higher efficiency than that possible with a man-made machine [4]. In order to create biomimetic systems, polymer gels have been employed due to their reversible size and shape change, thereby realizing the motion by integrating the deformation on a molecular level. The contractile collagen fiber prepared by Katchalsky *et al.* are the earliest man-made example of gel actuators [5, 6]. Later on, Osada *et al.* and others developed several gel machines based on synthetic polymers [7–13]. Recently, various types of peptide-based gels as well as hybrid gels have been developed [14–18]. The specific properties of these gels might be beneficial to constructing soft actuators based on the concept of molecular machines [19, 20].

Polypeptides from living organisms are attractive materials because of their high functionality, such as enzyme activity, ability of self-organization, or selective recognition. Some groups have constructed devices by depositing or arranging polypeptides on substrates. For example, thin films composed of bacteriorhodopsin [20] have been successfully used as photoelectric devices, and thin films of glucose oxidase as glucose-response biosensors [21]. Actin-based metallic nano-wires and actuators were also constructed recently [22–25].

Furthermore, various kinds of motor proteins, such as actin myosin [26–28] or microtubule kinesin [29], as well as other proteins that generate rotational motion like F1-ATPase [30], have been used as micro-motors [31]. Thus micro-machines and micro-devices driven by bio-molecules have been received considerable attention. However, most of these devices have exploited only in one aspect of biological function, partly because the polypeptides were immobilized or arranged two-dimensionally on the surface of substrate. By using the chemical cross-linking technique, polypeptides could have a highly ordered structure such as three-dimensional gels.

Actins and myosins are major components of muscle proteins and play an important role in dynamic motion of animals that is caused by the molecular deformation using the chemical energy released by hydrolysis of ATP. Recently, we have found that F-actins can be self-assembled into large bundles in the presence polycations through polymer complex formation. These polycation-actin complexes, several tens of times the length of native actin filaments, move along a chemically cross-linked myosin fibrous gel with a velocity as high as that of native actins, by coupling to ATP hydrolysis [22–24, 32]. This result indicates that muscle proteins can be tailored into desired shape and size without sacrificing their bioactivities, and can be used as biomaterials.

## 2. Actin gel formed from polymer-actin complexes [32]

Under physiological conditions, globular actin (G-actin) monomers are self-assembled into linear filaments (F-actin). F-Actins also assemble into parallel bundles or form a cross-linked network in the presence of actin linker proteins. It is believed that specific linker proteins are responsible for the morphology of actin assemblies [33] However, it has been reported that the various morphologies of actin bundles such as *Drosophila* bristle, nurse cell strut, stereocilia, and the acrosomal process are independent of the specific type of linker proteins. In addition, it has been reported that the binding of some proteins, including calponin, dystrophin, and MARKS peptide, to F-actins is due to electrostatic interaction between negatively charged F-actin and positively charged binding proteins without specific binding sites [34–36]. These facts indicate that the morphology of actin assemblies is determined not only by specific linker proteins but also by other factors such as the concentration of the linker proteins, environmental conditions and the kinetics of actin-linker protein interactions. Here, we employed a model system consisting of F-actin and synthetic cationic polymers to create assemblies with various morphologies.

**Figure 1.**
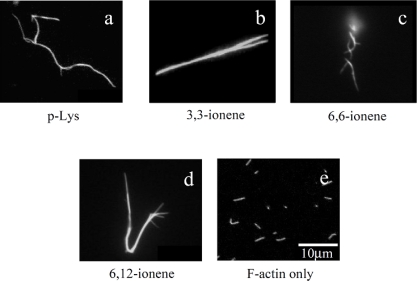
Fluorescence microscope images of polymer-actin complexes formed by mixing F-actin and various cationic polymers at room temperature. (a) p-Lys, (b) 3,3-ionene, (c) 6,6-ionene, (d) 6,12-ionene, (e) F-actin only. The molar ratio of ammonium cation of polymer to monomeric actin was kept constant at 30:1 for x,y-ionene polymers and 100:1 for p-Lys, which correspond to weight ratios of [3,3-ionene]/[actin]) 0.41 g/g, [6,6-ionene]/[actin]) 0.61 g/g, [6,12-ionene]/[actin]) 0.81 g/g, [p-Lys]/[actin]) 0.35 g/g. Actin concentration was 0.001 mg/mL. Taken from ref. [32] with permission.

Since the isoelectric point of actins is pH 4.7, F-actins are negatively charged in neutral buffers. Therefore, they have been assumed to form complexes with cationic polymers through electrostatic interaction. Figures 1(a)–(d) show some examples of fluorescence microscope images of polymer-actin complexes obtained by mixing F-actins with poly-L-Lysine (p-Lys) [Figure 1(a)] and *x,y*-ionene polymers, which have a -[-(CH_2_)_x_-N^+^Br^−^(CH_3_)_2_-(CH_2_)_y_-N^+^Br^−^(CH_3_)_2_-]_n_- structure [Figures 1(b)–(d)], for 120 min. The morphology of the complexes depends on the chemical structure of the polycations [32]. One can see that large filamentous, stranded and branched complexes of 20–30 μm in size are formed in the presence of p-Lys, 3,3– 6,6- and 6,12-ionene polymers and their morphological nature, both size and shape, are remarkably different from that of native F-actin [Figure 1(e)]. The relationship between the morphology of polycation-actin complex and the concentrations of components is discussed in Section 3.

**Figure 2.**
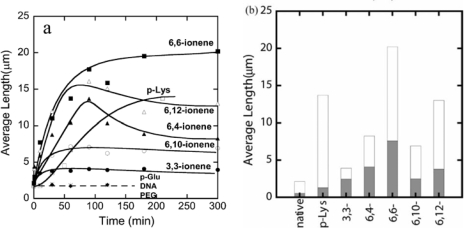
(a) Time courses of polymer-actin complexes growth. (•): 3,3-ionene-actin complexes, (▴): 6,4-ionene-actin complexes, (▪): 6,6-ionene-actin complexes, (∘): 6,10-ionene-actin complexes, (▵): 6,12-ionene-actin complexes, (□): p-Lys-actin complexes, (♦): p-Glu-actin complexes, (×): [DNA-actin complexes, (+): PEG-actin complexes. (b) Average length of polymer–actin complexes observed from fluorescence microscope images (white columns) and from transmission electron microscope (TEM) images (shade columns) at 210–300 min. The molar ratio of ammonium cation to monomeric actin was 30:1 for x,y-ionene polymers and 100:1 for p-Lys. The corresponding weight ratios were as follows: [3,3-ionene] /[actin]=0.41 g/g, [6,4-ionene]/[actin]=0.54 g/g, [6,6-ionene]/[actin]= 0.61 g/g, [6,10-ionene]/[actin]=0.74 g/g, [6,12-ionene]/[actin]=0.81 g/g, [p-Lys]/ [actin]=0.35 g/g, [p-Glu]/[actin]=0.36 g/g, [DNA]/[actin]= 0.77 g/g, [PEG]/[actin]=0.10 g/g. Actin concentration: 0.001 mg/mL. Taken from ref. [32] with permission.

The number-average length of fluorescence image of F-actins is 2.14 μm, with a standard deviation of 0.11 μm (average over 784 samples) in the F-buffer that is known to cause a transformation to the fibrous polymerized state of actin (F-actin) from the globular monomer actin (G-actin). However, polymer-actin complexes grow with time and reach as large as 5–20 μm within 1 or 2 h, which is about 2–10 times larger than that of native F-actin. p-Lys produces a large complex, whereas 3,3-ionene polymer gives the smallest complexes. These results imply that hydrophobicity and charge density of the ionene polymers are important in complex formation. In the case of 6, 4- and 6, 10-ionene polymer, after reached maximum, decrease in the average length of the complexes is observed. This is attributed to the compacting of the F-actins in the complexes. The effect of electrostatic interactions is proved, since no complexation was observed with negative controls such as p-Glu, DNA and neutral polymer, such as PEG. The average lengths of polymer-actin complexes are shown in Figure 2(a) (b).

Concerning the analysis of the lateral structure of the polymer-actin complexes, transmission electron microscopy (TEM) accompanied with negative staining technique was employed. Although the samples must be exposed to high vacuum in a dry state in this technique, the higher resolution offers advantage compared to analyzing from the images of fluorescent microscopes concerning such a precise structure. The TEM measurements show that the polymer-actin complexes are bundles that consist of 3–20 filaments [24]. The average width of the p-Lys-actin complex is 21.0 nm, with a standard deviation of 2.6 nm. Comparing with the native F-actins that have an average width of 12.1 nm with a standard deviation of 1.2 nm, p-Lys-actin complexes are only slightly thicker than that of the native F-actin with almost the same width scattering. 3,3-ionene complexes also showed a very thin and homogeneous wire-like morphology, showing an average width of 16.1 nm with a standard deviation of 1.7 nm.

**Figure 3.**
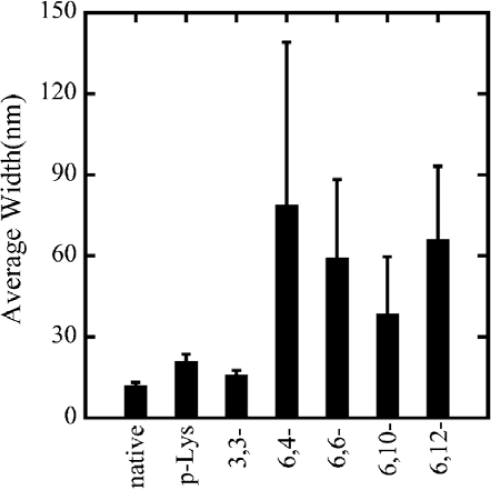
Average width of polymer–actin complexes obtained at 240 min by TEM images. Error bar means standard deviation. Taken from ref. [32] with permission.

In the case of *x,y*-ionene polymers, they form bundles of thin filaments with F-actin above certain critical polymer concentration that are dependent on each polymer. The effect of *x,y*- of the ionene polymers was observed in the width of complexes. Actin- 6,4-, 6,6-, 6,10-, and 6,12-ionene complexes have average widths of 79.0, 59.3, 38.7 and 66.1 nm, with a standard deviation of 60, 29, 21, and 27 nm, respectively (Figure 3). From the large scattering in the width of actin-6,4-, 6,6-, 6,10-, and 6,12-ionene complexes, the morphology of complexes seems to have randomness quantitatively. Indeed, a ring-shaped complex (nano-ring) is observed in a 6,6-ionene-actin complex occasionally[32].

## 3. Polymorphism of actin complexes [37]

**Figure 4.**
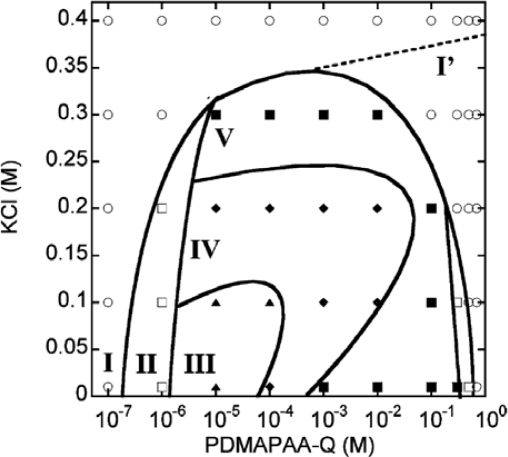
Phase diagram for the morphology of polymer-actin complexes, in which the phase behavior is summarized as a function of PDMAPAA-Q concentration CP and KCl concentration CS for a constant actin concentration CA = 0.01 mg/mL (2.32 × 10-7M). ∘, F-actins; □, coexistence (polymer-actin complex and native F-actins); ▴, cross-linked structure dominant phase; , branched structure dominant phase; and ▪, parallel bundle dominant phase. The dotted line shows the possible borderline between the native F-actin and F-actin with charge inversion. Taken from ref. [37] with permission.

**Figure 5.**
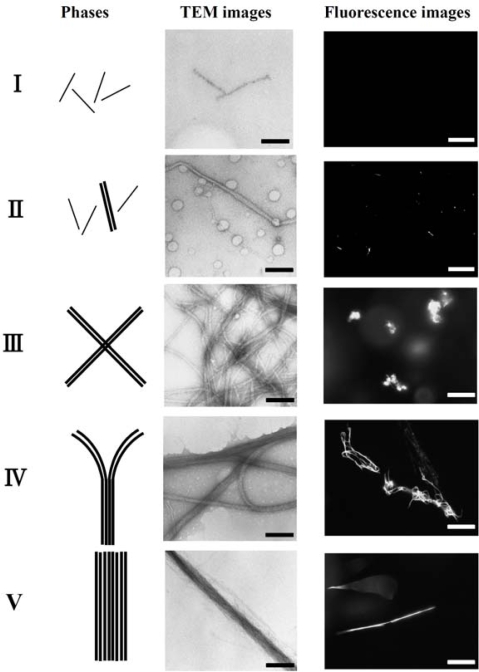
Typical morphologies of polymer-actin complexes in phases I, II, III, IV, and V as observed by TEM images and fluorescence images. Scale bars present 200 nm for TEM images and 25 μm for fluorescence images. Taken from ref. [37] with permission.

In order to investigate the polymorphism, the complexation of F-actin with poly-*N*-[3-(dimethyl-amino)propyl]-acrylamide(PDMAPAA-Q), which has positive charges on its side chain, were further investigated systematically. The polymer-actin complexes exhibit a rich polymorphism in a wide range of the polycation concentration (*C*_P_) and KCl salt concentration (*C*_S_), as elucidated by the fluorescent analysis and TEM analysis, which show micro- and nano-scale images, respectively. There are five characteristic phases in the *C*_P_-*C*_S_ phase diagram (Figure 4). Figure 5 shows the fluorescent images and TEM images for the polymer-actin complexes in the five phases. In phase I, F-actin does not grow. In phase II, we observe the coexistence of native F-actin and polymer-actin complexes. In the coexistence phase (II), the fraction of native F-actins increases with the increase of *C*_S_. The borderline between phases I and II shifts to a higher *C*_P_ with the increase of *C*_S_. This can be explained by screening effect of salts on the electrostatic interaction between F-actins and polycations. As *C*_P_ and *C*_S_ increase, the F-actins form complexes with polycations and exhibit various structures. The polymer- actin complexes evolve from the cross-linked structure dominant phase (III), to the branched-structure dominant phase (IV), and then to the parallel-bundle dominant phase (V) as shown by the TEM images in Figure 5.

The fluorescent images in Figure 5 show that the polymer-actin complexes of the cross-linked structure dominant phase (III) are in compact globule state, whereas those of the parallel bundle dominant phase are in the extended state (V). The globule size of 15–20μm is attributed to the persistence length of F-actin, which is assumed as ca. 10μm. The morphological change of polymer-actin complexes from the compact globule state to the extended bundle state is due to the increase of bending rigidity that increases with the thickness *D* of actin bundles, varying as *D* [38, 39].

## 4. Anisotropic nucleation growth mechanism for actin bundle [40]

A model system consisting of F-actin and p-Lys is used to investigate the physical origin of the well–defined thickness *D* and length *L* of the actin bundles. This system reveals that *D* growth is nearly completed in the initial stage of bundle growth, while dramatic *L* growth starts later on, after completion of *D* growth. Additionally, *D* decreases with the increase in the polycation mediated attraction between F-actins but is hardly influenced by the actin concentration (*C*_A_), while *L* increases with the increase of *C*_A_. From these results, an anisotropic nucleation-growth model is proposed, in which *D* is determined by the critical nucleus size *D*_0_, while the length *L* is determined by free actin concentration relative to nucleus concentration.

Here, the factor to determine *D* in detail is discussed. The free energy, Δ*G* of forming bundles can be expressed as Δ*G* = Δ*G_vol_* + Δ*G_Surf_* = Δ*gV* + *γS*, where Δ*g* is the energy gain due to bundle formation of F-actins per volume, *V* is the volume of actin bundles, *γ* is the surface energy, and *S* is the surface area of actin bundles. For the nucleation-growth process, spontaneous growth occurs only when stable nuclei are formed, which occurs when the favorable volume term Δ*G_vol_* surpasses the unfavorable surface term Δ*G_Surf_*. It is presupposed that the bundle is a cylindrical object with a length *L* and a thickness *D*. The free energy Δ*G* (*D, L*) for the formation of the bundle is:

(1) $$\begin{array}{ll} \Delta G=-\frac{\pi D^{2}L}{4}\Delta g+\frac{\left(\pi D^{2}+2\pi DL\right)}{2}\gamma & \approx -\frac{\pi }{4}(\Delta gD^{2}L-4\gamma DL) \end{array}$$

where it is assumed that for a newborn nucleus, *D*≪ *γ*/Δ*g* ≪ *L*. The critical thickness is *D*_0_is determined by the free energy barrier ΔG* where Δ*G* is maximum. From $\frac{\delta \Delta G}{\delta D}=0$, we have

(2) $$D_{0}\approx \frac{2\gamma }{\Delta g}$$

(3) $$\Delta G^{*}\approx \frac{\pi \gamma^{2}L}{\Delta g}$$

Eq. (2) and (3) indicate that *D*_0_ and ΔG* are reciprocally proportional to the energy gain per volume for bundle formation Δ*g*. This anisotropic growth of actin bundles is originated from the rod-like polyelectrolytes nature of F-actins. Therefore, this model not only provides insight into physical origins of how the growth of cellular actin bundles is determined but also can be broadly applied to the self-assembly of rod-like polyelectrolytes.

## 5. Three dimensional structure of actin bundles formed with polycations [41]

In Sections 2 and 3, the thickness *D* of actin bundle is evaluated by using TEM projection images, which smear the cross-sectional structure information. However recently developed transmission electron microtomography (TEMT) allows to obtain the three dimensional structure image of actin bundles.

**Figure 6.**
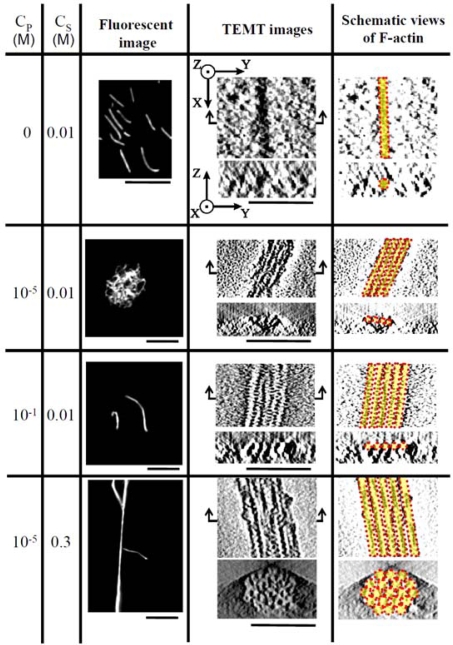
Fluorescence images and cross-sectional images of the actin bundles formed with PDMAPAA-Q at various concentrations of polymer (C_P_) and KCl (C_S_) obtained by fluorescence microscopy and transmission electron microtomography (TEMT). Arrows along the x-y plane of the TEMT images represent the positions of the images taken along the y-z plane. The fluorescence and cross-sectional images presented are drawn to scales of 10 μm and 50 nm, respectively. Taken from ref. [41] with permission.

**Figure 7.**
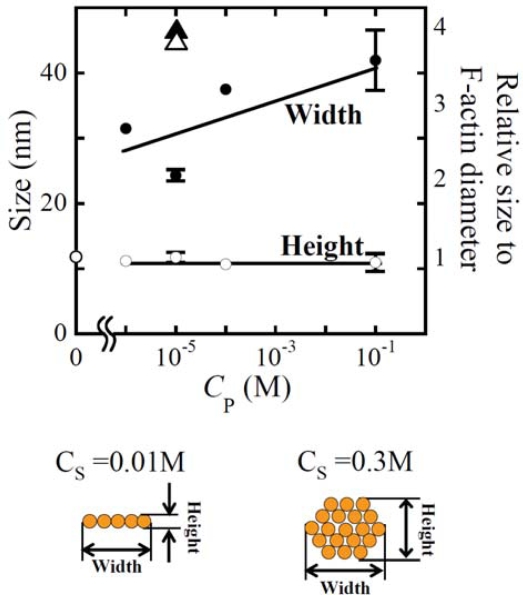
The width (closed symbols) and height (open symbols) of the actin bundles formed at various PDMAPAA-Q concentration (C_P_) and KCl concentration (C_S_). (•, ∘), C_S_ = 0.01 M; (▴,▵), C_S_ = 0.3 M. The width and height, defined in the illustration, are measured along the guide line in the schematic views of Figure 1. The size of the bundle formed at C_P_ = 10^−5^ M, C_S_ = 0.01 M and C_P_ = 10^−1^ M, C_S_= 0.01 M was the average of two and three samples, respectively. Other data were measured for one sample. The diameter of F-actin is 11.8 nm, as determined from Figure 1, which corresponds well with our previous result [32]. The relative size to the F-actin diameter, which means the ratio of the bundle width or height to the F-actin diameter, is also displayed in the figure as the right vertical axis. Taken from ref. [41] with permission.

Figure 6 shows the fluorescent and TEMT images of actin bundles formed with PDMAPAA-Q at various *C*_S_ and *C*_P_. In the absence of polycations, F-actins at a concentration of 0.01 mg/mL are present as single filaments of thickness 10 nm; the filaments exhibit a polydisperse length distribution of 1–10 μm, with an average of approximately 5 μm. The actin bundles show a ribbon-like cross-sectional morphology with lateral packing of the actin filaments in the presence of polycations at a low *C*_S_. The cross-sectional morphology changes to cylindrical with hexagonal packing at a high *C*_S_. The width and height of the bundles formed at various *C*_P_ and *C*_S_ are summarized in Figure 7. At a constant *C*_S_ of 0.01M, the width increases with an increase in *C*_P_, while the height barely depends on the *C*_P_, showing a value that is the same as that of F-actin. In contrast, at a constant *C*_P_ of 10^−5^ M, both the width and height increase with an increase in *C*_S_ from 0.01 to 0.3 M (triangular symbols). The fluorescent image in Figure 6 reveals that the actin bundles undergo morphological changes from a compact globule structure to an extended structure. These morphological changes might partially be attributed to the cross-sectional changes in the structure of actin bundles form a ribbon-like structure that is flexible to a cylindrical structure that is rigid.

Thus, cross-sectional morphology of actin bundles depends on the ionic strength but is insensitive to the concentration of polycation. The anisotropy in the cress-sectional structure of actin bundles may originate from an anisotropic electrostatic interaction between F-actins. At present, no satisfactory explanation on how this anisotropy is induced has been given. According to the anisotropic nucleation growth model, which states that *D* is determined at the nucleation stage, the anisotropy in the lateral direction should occur in the initial nucleation stage when F-actins form nuclei mediated by polycations [40].

## 6. Oriented myosin gel formed under shear flow [22, 23]

**Figure 8.**
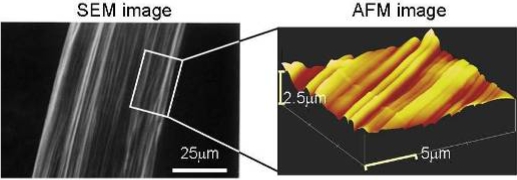
The orientation of myosin fibers in the gels was analyzed by scanning electron microscopy (SEM) and atomic force microscopy (AFM).

Chemically cross-linked myosin gel with its oriented filament array can be obtained by reacting scallop myosin at pH 7.0 using transglutaminase (TG) under shear flow-induced stretching. The oriented myosin gel is semi-transparent, showing a swelling degree of *ca*. 100, and a Young modulus of 190 Pa in the oriented direction, which is more than two times larger than that of the myosin gel prepared without stretching. The orientation of myosin fibers in the gels was analyzed by scanning electron microscopy (SEM) and atomic force microscopy (AFM) (Figure 8). From the images, distinct bundles of regularly oriented filaments ca. 1.5 μm in diameter were observed, indicating that the rodlike myosin molecules are self-organized with orientation to form a hierarchical structure. The molecular orientation within the filaments was confirmed by the strong IR dichroism of carbonyl absorption at 1600 cm^−1^, which could not be observed in the absence of stretching. The chemically cross-linked myosin gel shows an ATPase activity as high as that of native myosins in the presence of 0.5 w/w native actin. Myosin gels cross-linked by other cross-linking agents, such as glutaraldehyde and 1-ethyl-3-(3-dimethylaminoprolyl)carbodiimide, also showed an ATPase activity, though not as high as those cross-linked by TG. The motion of these myosin gels will be discussed in next section.

## 7. Motility assay of F-actin on oriented myosin gel [23]

F-actins showed a preferential motion along the axis of oriented myosin gel as elucidated by the degree of anisotropy (D.A.), which is defined as the ratio of the square-root average velocity in the fiber direction to that perpendicular to the fiber direction.

(4) $$D.A=\frac{\bar{x}}{\bar{y}}$$

where,

(5) $$\bar{x}=\sqrt{\sum_{i=1}^{N}\frac{x_{i}^{2}}{N},}\;\bar{y}=\sqrt{\sum_{i=1}^{N}\frac{y_{i}^{2}}{N}}$$

The D.A. measured on the non-oriented myosin gel was 1.1 (average over 66 samples), and that on the oriented myosin gel was 1.7 (average over 91 samples). The mean velocity on the non-oriented myosin gel was 0.69 μm/s with a standard deviation of 0.24 μm/s, while that on the oriented myosin gel was 0.83 μm/s with a standard deviation of 0.30 μm/s. Thus, F-actin filaments prefer to move along the axis of the oriented myosin gel with an increased velocity.

## 8. Motility assay of polymer-actin complex gel [22]

The p-Lys-actin complex can be cross-linked to obtain a stable structure. The complex gel cross-linked with TG also shows a high motility on the oriented myosin gel in spite of its large dimension.

Here, it should be noted that if no cross-linking was performed on the polymer -actin complexes, disassembling of them into F-actins is observed. The p-Lys-actin complex gels move preferentially along the axis of the oriented myosin gel almost without path deviations. Figure 9 shows the sequential fluorescent image (a) and velocity distributions (b) of p-Lys-actin complex gels on the oriented myosin gel as a function of the individual filament size. The p-Lys-actin complex gels, about four times larger than that native F-actin, move with an average velocity of 1μm/s, almost the same as that of native F-actins on the oriented myosin gel (opened circles). Some of the p-Lys-actin complex gels move as fast as 2.0 μm/s. In addition, D.A. of the p-Lys-actin complex gels was 2.2 (average over 38 samples) on the oriented myosin gel, which was higher than that of the native F-actin (D.A. = 1.7), indicating an enhanced directional preference along the axis of the oriented myosin gel. Thus, despite its increased mass, the p-Lys-actin complex gels, several tens to hundreds times the volume of the native F-actins, move on the covalently cross-linked myosin gel, with an increased velocity. This is rather surprising, since the interaction between the myosin gel and the actin gel can only occur at the two-dimensional interface and due to cross-linking a considerable number of actin and myosin molecules are not involved in the sliding motion [22]. These results indicate that actins can be tailored into the desired shape and size without sacrificing their bioactivities by using complex formation with synthetic polymers.

## 9. Polarity of the actin in complexes [24]

On a glass surface where myosin molecules are simply immobilized, F-actin is known to show motility. We found that all these polymer-actin complex gels, including those from p-Lys, PDMAPAA-Q, and *x,y*-ionene bromide polymers (x=3or 6; y=3,4,or10), also exhibit motility and the velocity of the complexes were comparable to that of native F-actin (0.77±0.32μm/s) [24].

**Figure 10.**
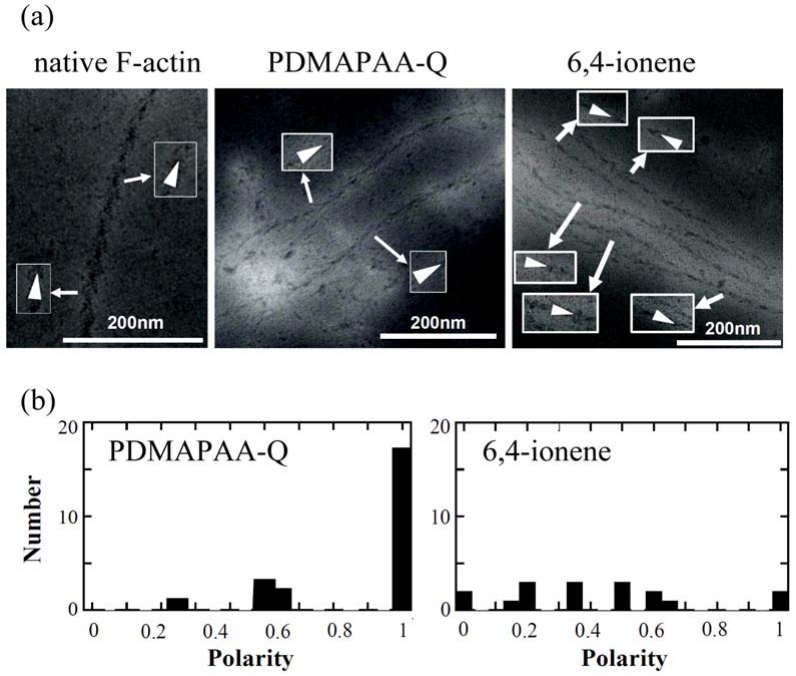
(a) Polarity of polymer-actin complexes decorated with HMM. White arrows indicate the direction of arrowhead structures of decorated filaments. (b) Histograms of complex polarity distributions. Cited from ref. [24] with permission.

The polarity of polymer-actin complexes, which is considered to be essential in the sliding motion of polymer-actin complex gels, has not been completely clarified yet. This question is important for understanding the cooperative motion by the actin assembly. An arrowhead-like pattern can be observed under transmission electron microscopy (TEM) when heavy meromyosin (HMM) was decorated on native F-actin [Figure 10(a)], indicating that F-actin has a well-defined polarity by self-organization. The pointed end of arrowhead and the opposite end of arrowhead are called the p-end and b-end, respectively.

To evaluate the polarity of actin complexes, we also attempted to decorate the actin complexes with HMM. Figure 10(a) shows some examples of TEM images of HMM-decorated PDMAPAA-Q-actin complexes and 6,4-ionene-actin complexes. Different from native F-actin that is a single strand, the polymer-actin complexes are bundles that consist of 3–20 filaments. Arrowhead structures within a filament of the bundle pointed in the same direction, although some defects are observed occasionally. However, arrowhead directions of filaments within a bundle are not completely the same. The complex polarity *P* defined by Eq. (6) was estimated.

(6) $$P=|n_{1}-n_{2}|/|n_{1}+n_{2}|$$

Here n_1_ and n_2_ are the number of filaments pointed in the opposite directions.

The average polarity of the actin complex is shown in Table 2. As seen in this data, polarity depends on the chemical structure of polycations, and PDMAPAA-Q-actin complexes show the highest polarity as 0.89 (average over 23 samples), while 6,4-actins show the lowest value as 0.42 (average over 17 samples). Thus, PDMAPAA-Q prefers to form actin filament bundles having a uni-polarity.

Among all these polymer-actin complexes, the PDMAPAA-Q-actin complex, which has the highest polarity, also shows the highest motility, with a velocity of 1.3 μm/s. Dendritic complexes, which are occasionally observed when F-actin is mixed with 6,4- ionene, did not exhibit a translational motion but instead migrate around their barycentric position. When the velocity evaluated at 3.3s intervals is plotted agains the polarity, a linear relationship is observed, i.e. the velocity of the complexes is proportional to the polarity. These results suggest that the polarity of the polymer-actin complex is essential in producing a high motility. It is known that a native F-actin always moves toward one direction without moving back to the opposite direction. The direction of the F-actin motion is associated with its polarity. For the polymer-actin bundles that are assembly of F-actin, the whole polarity is determined by the polar direction of F-actin. If the F-actin filaments assemble in an anti-parallel way, the whole polarity is cancelled and leads to no sliding motion. This explains why the correlation between polarity and velocity is observed. However, the difference between the velocity of native F-actin and that of the polymer-actin complex gels with a polarity close to 1 (p-Lys-actin and PDMAPAA-Q-actin) cannot be explained only in terms of polarity. The higher velocity observed for the complex gels should, therefore, be attributed to two possible factors: 1) the arrowhead structure that depends on the structures of polycations, and 2) the bundle formation. If the change of the arrowhead structure is attributed to the change of the helix pitch of an actin filament, an elongation of arrowhead pattern will cause the extensions of helix structure of the actin filament. In consequence, the elasticity of the actin filament might increase and cause the effective dynamic-interaction with myosin which favors the motion. On the other hand, by forming an actin bundle, the bending fluctuation is eliminated, and this also leads to an effective integration of driving-forces from each myosin molecules.

To elucidate the randomness of the motion, we also investigated the trajectory of the motion [24]. From this characterization, it is found that all polymer-actin complexes show more translational motion than that of native F-actin. Because polymer-actin complexes are bundles formed from actin filaments, they are less flexible than native F-actin. The less random motion of polymer-actin complexes is attributed to the less structural flexibility.

## 10. Spatiotemporal control of actin assembly [42, 43]

**Figure 11.**
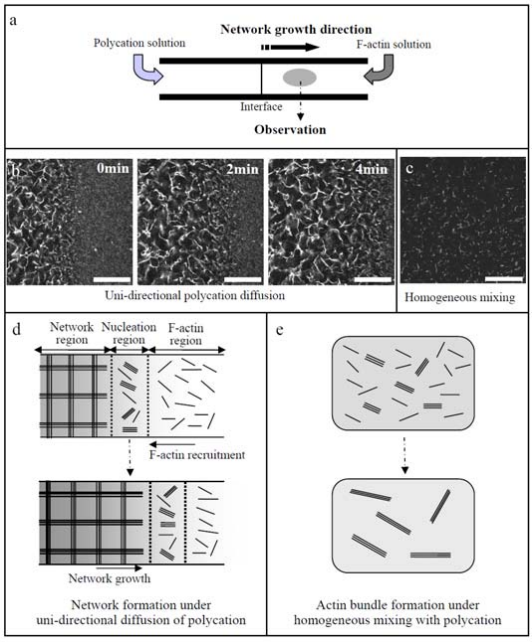
(a): Schematic illustration of microchamber for the uni-directional diffusion of polycation to F-actin solution. (b): Time-lapse images of actin network formation after the first 10 min of the uni-directional diffusion of PDMAPAA-Q (C_P_ = 0.1 M) to F-actin solution (C_A_ = 0.01 mg/mL in microchamber. (c): Actin bundles formed by homogeneous mixing of F-actin solution (C_A_ = 0.01mg/mL) with PDMAPAA-Q solution (C_P_ = 0.1 M) and then incubating for 60 min. (d, e): Schematics of actin network formation under uni-directional diffusion of polycations (d), actin bundles formation under homogeneous mixing with polycations (e). Black rods and the intensity of gray background represent F-actins and polycation concentration, respectively. Scale bar presents 50*μm*. Cited from ref. [42] with permission.

In Sections 7 and 8, the motility system composed of the biomolecular motor (actin/myosin) assemblies was described. To enhance their potential application, introduction of hierarchical structure into the assemblies is inevitable. Within living cells, in order to organize their body with ordered structure, the bio-molecule (actins or other motor proteins) assembly process is spatially controlled by the localization of binding proteins, and also temporally regulated by the coupling of the binding proteins to signal molecules [44–46]. Intrinsic polarity of cells leads the binding proteins and signal molecules to exhibit a strong localization within a cell. It can be demonstrated that under asymmetric conditions by unidirectional diffusion of polycations (PDMAPAA-Q) to F-actin solution F-actins are effectively assembled into a globally linked network even at a low actin concentration that only small actin bundles are formed under homogeneous mixing of F-actins with polycations (Figure 11). This fact indicates that the asymmetric spatial distribution of polycations is essential for the architectural variety of F-actin bundles.

**Figure 12.**
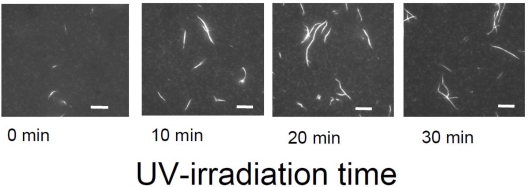
Fluorescent micrographs of the photo-induced bundle formation of F-actin/photoresponsive polycation complex, where [F-actin]/[photoresponsive polycation] is fixed at 1.0 g/g, and [F-actin] = 5×10^−3^ mg/mL. The scale bars are 10 μm.

**Figure 13.**
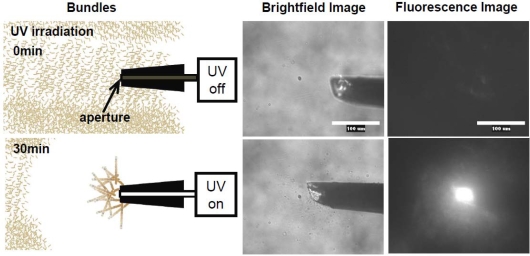
Photo-induced local formations of actin complex at the UV photon emitting tip-edge grass capillary connected to the optical fiber. In the brightfield image of fluorescent microscope, the block object is the tip-edge of grass capillary. The scale bar is 100 μm. The observation was performed before and after the local UV irradiation in 30 min.

Meanwhile, temporal control of the actin assembly formation can be achieved by applying a photoresponsible polycation composed mainly of DMAPAA-Q and partially of photochromic molecules (triphenylmethane leucohydroxide) that can be cationized in aqueous solution by ultraviolet (UV) irradiation. As shown in Figure 12, the photo-induced actin assembly formation is observed following UV irradiation. The localization of UV irradiation by using an optical tip made of a glass capillary enables to control the formation of F-actin/photoresponsive polycation complex spatiotemporally. As shown in the fluorescence micrograph in Figure 13, prior to UV irradiation, F-actin is in its native state. Following UV irradiation through the glass capillary tip, the large aggregated structure of the F-actin/photoresponsive polycation complex is observed only at the tip. Thus, we can remotely trigger and confine the formation of F-actin assemblies to any location of interest within a complex system. This technique will provide a method to create F-actin assembly with highly ordered structure by 3D drawing system with UV light.

## 11. Conclusions

Biopolymers including F-actin have a lot of potential as functional materials in biotechnology and biomaterials science. It has been revealed that actin and myosin vary their functionality depending on the orientation within the complexes. In the case of myosin, it has been found to have self-assembly ability. On the other hand, actin has shown an ability to form a functional complex with polycations by electrostatic interaction. Moreover, the cross-linked actin complex gels also have shown motility on the myosin gels. The motility of actin complex gels depends on the structure that is determined by the conditions such as the concentrations or the structures of the polycations. This may also be applicable for the other functional biopolymers. To develop more functional biomaterials hereafter, how to orientate the functional domain or the polarity, may become the most significant issue. The basic analysis and technical development to form and stabilizing the biopolymers by cross-linking may contribute to promote the possibility of the biopolymers as functional materials in biotechnology and biomaterials science.

On the other hand, many studies on the mechanism of muscle contraction have been performed both on macroscopic level using the physiological technique and on molecular level using optical microscope, optical tweezers, and etc. However, the approach from a mesoscopic level, from between micro-level and macro-level, has not been studied completely. New architectures made from muscle protein will also provide a mesoscopic insight into the mechanism of muscle contraction including the role of hierarchical structure and the mechanism of cooperative sliding motion

## Figures and Tables

**Figure 9(a).**
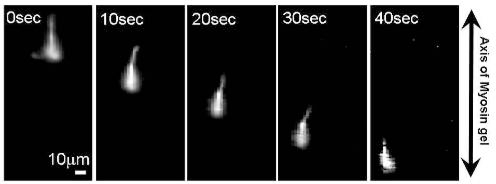
The sequential fluorescent images of p-Lys-actin complex gels on the oriented myosin gel

**Figure 9(b).**
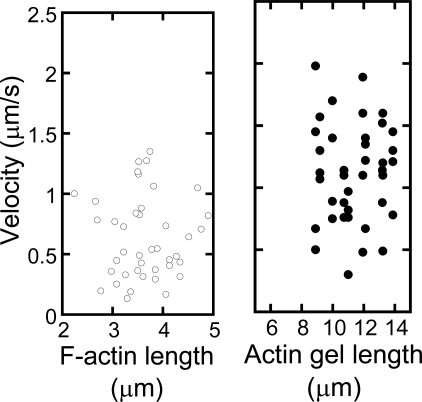
Individual velocities of native F-actins (left; opened circle) and p-Lys-actin gels (right; closed circle) of various length on the oriented myosin gel.
